# Cationic Metal–Organic Framework-Based Mixed-Matrix Membranes for Fast Sensing and Removal of Cr_2_O_7_^2−^ Within Water

**DOI:** 10.3389/fchem.2022.852402

**Published:** 2022-02-28

**Authors:** Shuyun Zhang, Heqi Zheng, Yu Yang, Guodong Qian, Yuanjing Cui

**Affiliations:** Laboratory of Silicon Materials, Cyrus Tang Center for Sensor Materials and Applications, School of Materials Science and Engineering, Zhejiang University, Hangzhou, China

**Keywords:** metal–organic frameworks, mixed-matrix membranes, removal, fluorescence sensing, Cr_2_O_7_^2^

## Abstract

Considering that metal–organic framework (MOF)-polymer mixed-matrix membranes (MMMs) can overcome the drawbacks of intrinsic fragility and poor processability of pure-MOF membranes, we designed MOF-based MMMs for efficient removal and fast fluorescence sensing of heavily toxic ions within water systems simultaneously. In this work, a series of MOF-based MMMs are prepared by mixing a hydrolytically stable cationic [Eu_7_ (mtb)_5_(H_2_O)_16_]·NO_3_ 8DMA·18H_2_O (denoted as Eu-mtb) MOF material into poly (vinylidene fluoride) with high loadings up to 70%. The free volume at the interface between the polymer and Eu-mtb particles, combined with the permanent porosity and uniform distribution of Eu-mtb particles, enables these MMMs to show fast enrichment of Cr_2_O_7_^2-^ from solutions and consequently have a full contact between the analyte and MOFs. The developed Eu-mtb MMM (70wt% loading) thus shows both efficient removal and exceptional fluorescence sensing of Cr_2_O_7_^2-^ in aqueous media. The overall adsorption capacity of the Eu-mtb MMM (70 wt% loading) for Cr_2_O_7_^2-^ reaches up to 33.34 mg/g, which is 3.4 times that of powder-form Eu-mtb. The detection limit of the Eu-mtb MMM (70 wt% loading) for Cr_2_O_7_^2-^ is around 5.73 nM, which is lower than that of the reported powder-form Eu-mtb. This work demonstrates that it is feasible to develop flexible luminescent MOF-based MMMs as a significant platform for efficient removal and sensitive sensing of pollutants from water systems simultaneously.

## 1 Introduction

Heavy metal pollution has become a severe environment threat around the world. Hexavalent chromium ions, especially Cr_2_O_7_^2−^, has been widely employed in various industrial applications, such as metallurgy, pigment production, leather tanning, electroplating, and other relevant fields (Desai et al., 2016; Lai et al., 2018; Li et al., 2018). According to the U.S. Environment Protection Agency (EPA), Cr_2_O_7_^2−^ is classified as Group “A” human carcinogen, which can accumulate in living organisms, leading to a series of health problems such as aberration, gene mutation, and cancer (Zhang et al., 2015; Shen et al., 2018; Zou et al., 2018). Therefore, it is of vital importance to explore an effective method for capturing and detecting Cr_2_O_7_^2−^ from water systems simultaneously. Up to now, a variety of adsorbents have been widely employed to remove Cr_2_O_7_^2−^ from aqueous solution, including ACs (Cheng et al., 2016), layered double hydroxides (LDHs) (Lei et al., 2017), and resigns (Bhatti et al., 2014), but these methods show slow sorption kinetic, inferior selectivity, and low stability. So far, the traditional detection of Cr_2_O_7_^2−^ mainly depends on instrumental methods, such as inductively coupled plasma mass spectrometry (ICP-MS), atomic absorption spectroscopy (AAS), and electrochemical analysis (Araujo Barbosa et al., 2017; Bansod et al., 2017; Jiang et al., 2017; Wang et al., 2021), which are often high-cost and time-consuming. As a new class of sensing technique, fluorescence sensing has the advantages of real-time monitoring with better sensitivity, faster response time, lower cost, and less pretreatment of samples (Wu L. et al., 2020; Chen et al., 2021; Levine, 2021), which prompts the research of removing and detecting Cr_2_O_7_^2−^ simultaneously by developing a porous material with adsorption and fluorescence characteristics.

Metal–organic frameworks (MOFs), also known as the coordination polymer, are a promising class of porous materials constructed through the self-assembly of organic linkers and metal ions/clusters (Cui et al., 2018; Esrafili et al., 2020; Hu et al., 2020; Cao et al., 2021; Lv et al., 2021; Wu et al., 2021). Due to the merits of large surface areas, tunable structures, and excellent stability, MOFs have been widely employed in various applications, such as gas storage and separation, sensing, catalysis, and biomedicine (Wang et al., 2018; Zheng et al., 2018; Wu S. et al., 2020; Cheng et al., 2020; Hu E. et al., 2021; Hu M.-L. et al., 2021; Esrafili et al., 2021; Li et al., 2021; Zhou et al., 2021). Recently, several powder-form MOFs have been explored to remove and detect Cr_2_O_7_^2−^ from aqueous water simultaneously (Lin et al., 2017; Liu et al., 2019). However, it is noteworthy that only a part of MOFs can interact with analytes because of the difficulty of dispersing powder samples uniformly into the Cr_2_O_7_^2−^ solution, leading to the unsatisfied adsorption capacity and insufficient sensing sensitivity. The fabrication of powder-form MOFs into membranes is considered to be a valid and simple method to solve these drawbacks. Comparing to the harsh growth conditions and intrinsic fragility of pure MOF membranes, the incorporation of MOF particles into the polymer matrix to fabricate MOF-based mixed-matrix membranes (MMMs) is proved to be more competitive for realistic contaminant removal and sensing applications (Zhang et al., 2012; Dong J. et al., 2020; Wu T. et al., 2020). The integration of these two components has the ability to combine the flexibility and processability of polymers with the excellent properties of MOFs (Zhang et al., 2017; Li et al., 2019; Sousaraei et al., 2019; Chen et al., 2020; Muthukumaraswamy Rangaraj et al., 2020). Furthermore, the uniform distribution of MOF particles without aggregation within the polymers is beneficial for the sufficient interactions between MOFs and analytes (Denny Jr and Cohen, 2015; Semino et al., 2016; Jiang et al., 2021).

However, to the best of our knowledge, only two MMMs have been developed for the fluorescence sensing of Cr_2_O_7_^2−^ in aqueous solution (Qin et al., 2021; Xu et al., 2021), MMMs for the removal and sensing of Cr_2_O_7_^2−^ simultaneously within water have not been discussed yet. [Eu_7_ (mtb)_5_(H_2_O)_16_]·NO_3_ 8DMA·18H_2_O (Eu-mtb, H_4_mtb = 4-[tris(4-carboxyphenyl)methyl]benzoic acid)) is a hydrolytically stable cationic MOF that has been reported to have the lowest detection limit for Cr_2_O_7_^2−^ (Liu et al., 2017). Therefore, in this work, Eu-mtb was selected to be incorporated into poly (vinylidene fluoride) (PVDF) to fabricate a series of Eu-mtb MMMs. A mass of Cr_2_O_7_^2−^ from water can be easily enriched inside the MMMs by virtue of the free volume at the interface between PVDF and Eu-mtb particles, permanent porosity of Eu-mtb, and the electrostatic interaction between Cr_2_O_7_^2−^ and the cationic framework. Thereupon, the sufficient interaction between Cr_2_O_7_^2−^ and the Eu-mtb framework is achievable due to the uniform distribution of Eu-mtb particles. The as-prepared Eu-mtb MMM (70 wt%) shows a highly remarkable removal efficiency and detection sensitivity toward Cr_2_O_7_^2−^ in aqueous media. The overall adsorption capacity of the Eu-mtb MMM (70 wt%) for Cr_2_O_7_^2-^ is 33.34 mg/g, which is 3.4 times that of the powder-form Eu-mtb. The detection limit of the Eu-mtb MMM (70 wt%) for Cr_2_O_7_^2−^ is calculated to be 5.73 nM, which is lower than that of the reported powder-form Eu-mtb. The combination of the enhanced removal and sensing properties with its processability and flexibility makes Eu-mtb MMMs (70 wt%) a promising candidate for practical applications.

## 2 Experimental

### 2.1 Materials and Chemicals

Starting reagents and solvents were purchased and used without further purification: 4-[tris(4-carboxyphenyl)methyl]benzoic acid (H_4_mtb, ≥98.0%, Jilin Chinese Academy of Sciences—Yanshen Technology Co., Ltd.), europium nitrate hexahydrate (Eu(NO_3_)_3_ྷ6H_2_O, ≥99.9%, Energy Chemical), poly (vinylidene fluoride) (PVDF, average Mw ∼534,000, Aldrich), and N,N-dimethylacetamide (DMA, 99.5%, Sinopharm Chemical Reagent Limited Corporation). Deionized water and ethanol were utilized throughout all experiments.

### 2.2 Synthesis of Eu-mtb

Eu-mtb was prepared according to the published literature with some modifications (Liu et al., 2017). Briefly, 30 mg of H_4_mtb (0.06 mmol), 69 mg of Eu(NO_3_)3ྷ6H_2_O (0.15 mmol), N,N-dimethylacetamide (DMA; 3 ml), and deionized water (6 ml) were mixed and ultrasonically dissolved. The mixture was then sealed in a 25-ml Teflon-lined stainless autoclave and heated at 90°C for 2 days and subsequently cooled to room temperature. White block crystals were collected after filtration and washing with DMA and ethanol several times.

### 2.3 Fabrication of MMMs

Eu-mtb MMMs were prepared according to the published literature with some modifications (Zhang et al., 2018). The pure polymer membrane was manufactured as follows: PVDF (0.15 g) was dissolved in DMF (1.9 ml) and stirred for 1 day to form a sticky solution. MOF-based MMMs with different loadings were manufactured by adding Eu-mtb into the above solution and stirring for another day to form a homogenous solution. Afterward, a certain amount of mixed solutions was then cast onto a glass plate by a scraper to fabricate a flat sheet membrane under ambient conditions. Soaking in deionized water led to fast delamination of the membranes. Delamination of the membranes in water is possibility attributed to swelling of the PVDF, leading to a morphological change at the membrane/substrate interface and resultant release. MMMs with different loadings of Eu-mtb (30, 50, and 70 wt%) were prepared with thicknesses around 30–40 µm.

### 2.4 Measurements and Analysis

Powder X-ray diffraction (PXRD) patterns were collected in the 2θ = 5–50° range on a Shimadzu XRD-7000 diffractometer with Cu K*α* radiation (*λ* = 1.542 Å) at room temperature. Scanning electron microscopy (SEM) and energy dispersive X-ray spectroscopy (EDX) analyses were conducted on a Hitachi S4800 field-emission scanning electron microscope with a HORIBA EMAX energy dispersive spectrometer. Excitation and emission spectra were taken with a Hitachi F-4600 spectrofluorometer at room temperature. UV-vis spectra were measured with a Hitachi U-4100 ultraviolet spectrophotometer at room temperature. All the experiments were performed at room temperature. All error bars represent standard deviations from three repeated experiments.

### 2.5 Adsorption Experiments

#### 2.5.1 Adsorption Kinetics

2.1 ml of mixed solutions were cast onto a glass plate to fabricate membranes. During the adsorption process, the as-prepared MMMs were used for the removal of Cr_2_O_7_^2−^ with the concentration of 10 ppm. The Cr_2_O_7_^2−^ water solutions (48 ml) containing the MMMs were mixed well with magnetic stirring for 6 h at 25°C. During the stirring period, 2 ml of the mixture was taken out and filtered by syringe filters (PTFE, 0.25 μm), and the residual concentrations of Cr_2_O_7_^2-^ in the supernatant liquid were evaluated by UV-vis absorbance (monitor at *λ* = 257 nm).

#### 2.5.2 Adsorption Isotherm

265 μL of dope solutions were cast onto a glass plate to fabricate membranes. To obtain the adsorption capacity, MMMs were dispersed in 10 ml of Cr_2_O_7_^2−^ water solutions with a known concentration between 10 and 600 ppm, respectively. The mixtures were stirred at 25°C for 24 h and then filtered by syringe filters (PTFE, 0.25 μm), and the residual concentrations of Cr_2_O_7_^2-^ were evaluated by UV-vis spectroscopy.

#### 2.5.3 Adsorption Selectivity

440 μl of dope solutions were cast onto a glass plate to fabricate membranes. MMMs were dispersed in 10 ml of Cr_2_O_7_^2−^ water solutions (10 ppm) containing an *n*-fold molar excess of disturbing anions such as chloride (Cl^−^), nitrate (NO_3_^−^), and iodide (I^−^) (*n* is equal to 0, 1, 5, and 10). The mixtures were stirred at 25 °C for 24 h and then filtered by syringe filters (PTFE, 0.25 μm), and the residual concentrations of Cr_2_O_7_^2−^ were evaluated by UV-vis spectroscopy.

The equations related to the adsorption experiments are presented in the Supplementary Material.

### 2.6 Luminescent Sensing Experiments

The flow-through method: 300 μl of mixed solutions were cast onto a glass plate to fabricate membranes, and the areas of 2 cm^2^ were cut from the films for sensing tests. MMMs *via* cut were then fixed enclosed in a 250 ml micro vacuum suction device, the sand core diameter of which is 2 cm. Aqueous solutions (10^–3^ M, 100 ml) of K_2_Cr_2_O_7_, Ca(NO_3_)_2_, Zn(NO_3_)_2_, Pb(NO_3_)_2_, Al(NO_3_)_3_, Na_2_SO_4_, NaNO_3_, NaI, CH_3_COONa, and NaCl were respectively driven through the MMMs with a vacuum pump, and the treated MMMs were used for luminescent measurements.

The soaking method: For the purpose of comparison, a sample of as-prepared MMMs was soaked in the aqueous solutions (10^–3^ M, 100 ml) of K_2_Cr_2_O_7_.

## 3 Results and Discussion

### 3.1 Preparation and Characterization of Eu-mtb MMMs

**FIGURE 1:**
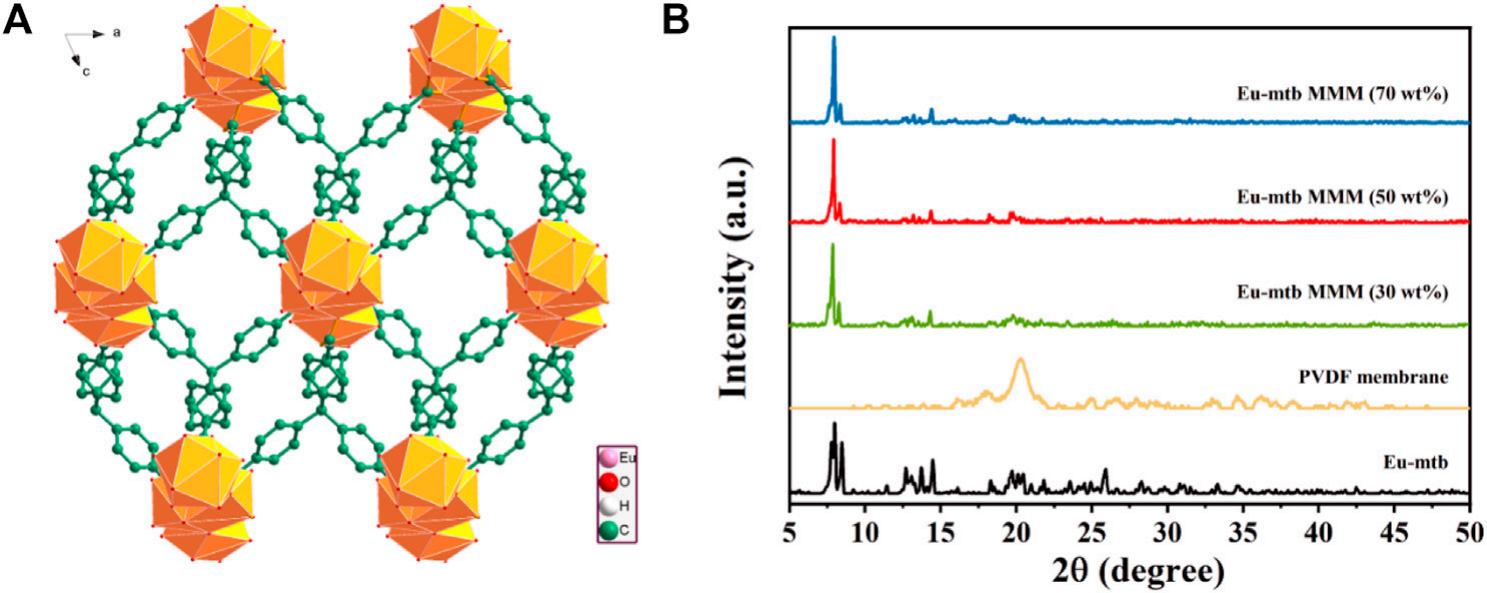
**(A)** 3D network structure of Eu-mtb viewed along the b axis. **(B)** PXRD patterns of Eu-mtb, PVDF membrane, and Eu-mtb MMMs.

As a kind of cationic MOFs, Eu-mtb was solvothermally synthesized from a mixture of europium nitrate hexahydrate (Eu(NO_3_)_3_·6H_2_O) and 4-[tris(4-carboxyphenyl)methyl]benzoic acid (H_4_mtb) in dimethylacetamide (DMA) and water solution. Eu-mtb is composed of 8-connected [Eu_3_O_25_]^+^ trinuclear core bound by mtb^4−^ ligands with four carboxylic groups to give the 3D structure having rhombic channels with a size of 7.2 × 6.4 Å^2^ (Figure 1A). The existence of charge-balancing NO_3_^−^ anions in the channels of Eu-mtb makes it possible for Cr_2_O_7_^2−^ in the aqueous solutions to enter into the pore surface of Eu-mtb through the anion-exchange process. The electrostatic interaction between the cationic framework and Cr_2_O_7_^2−^ is of great benefit for achieving efficient removal and sensitive fluorescence sensing of Cr_2_O_7_^2−^ within water simultaneously. The powder X-ray diffraction (PXRD) pattern of the resulted Eu-mtb is shown in Figure 1B, which was in good agreement with that reported in the previous literature (Liu et al., 2017). It is reported that MOFs with micro- or nanoparticles are often preferred to fabricate MOF-based mixed-matrix membranes (MMMs) because they can provide larger interfacial areas at the MOF-polymer boundary to allow closer integration (Dechnik et al., 2017). Therefore, scanning electron microscopy (SEM) images (Supplementary Figure S1) indicated that the size of the resulted Eu-mtb powder was around 10 μm, which is suitable for fabricating MMMs.

MMMs were fabricated by a drawdown coating (doctor-blading) process (Zhang et al., 2018). For the purpose of comparison, the pure polymer membrane was manufactured first as follows: PVDF (0.15 g) was dissolved in DMF (1.9 ml) and stirred for 1 day to form a sticky solution. MOF-based MMMs with different loadings were manufactured by adding Eu-mtb into the above solution and stirring for another day to form a homogenous solution. Afterward, a certain amount of mixed solutions was then cast onto a glass plate by a scraper to fabricate a flat sheet membrane under ambient conditions. Soaking in deionized water led to fast delamination of the MMMs, which is possibility attributed to the swelling of PVDF, leading to a morphological change at the MMM/substrate interface and resultant release. Eu-mtb-based PVDF hybrid membranes (denoted as Eu-mtb MMMs) with different weight loadings of Eu-mtb (30, 50, and 70 wt%) were obtained.

**FIGURE 2:**
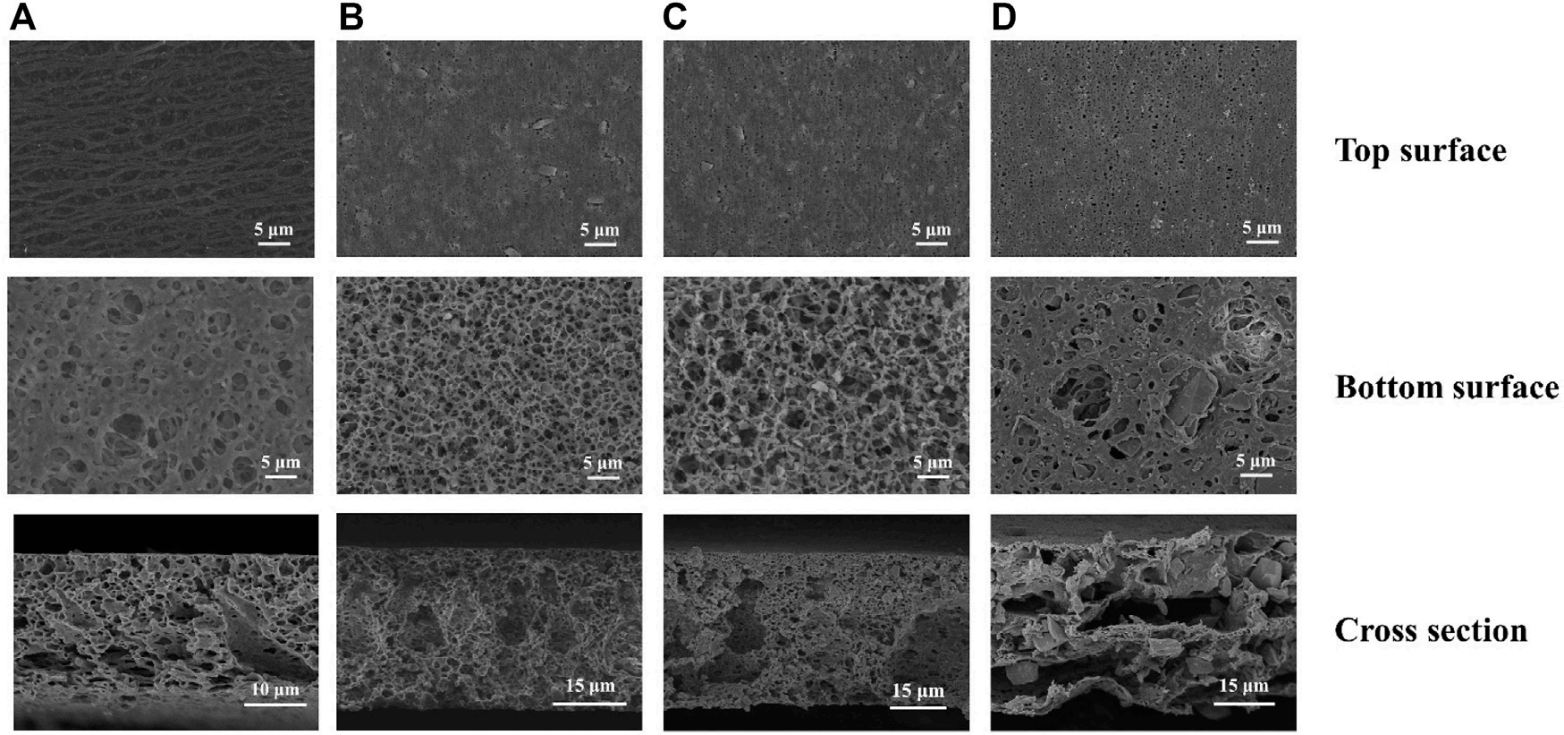
SEM images of **(A)** PVDF membrane, **(B)** Eu-mtb MMM (30 wt%), **(C)** Eu-mtb MMM (50 wt%), and **(D)** Eu-mtb MMM (70 wt%). Top, bottom, and cross-section surface.

The PXRD patterns of the resulted hybrid membranes are shown in Figure 1B. The broad peaks of the pure PVDF membrane are attributed to its amorphous properties. As the loading of Eu-mtb increases, these PVDF peaks decrease significantly, whereas the characteristic peaks of Eu-mtb appear and become predominant, which indicates that the crystallinity and structural features of Eu-mtb are maintained well in the MMMs. The SEM images of prepared Eu-mtb MMMs with different weight loadings (30, 50, and 70 wt%) are exhibited in Figure 2. Eu-mtb MMMs show morphologies similar to that of the pure PVDF membrane, demonstrating the incorporation of Eu-mtb particles causes almost no damage to the structural integrity of the polymer membrane. The Eu-mtb particles remain intact and are uniformly combined with the polymer binder without obvious aggregation.

Energy dispersive X-ray spectroscopy (EDX) was employed to characterize the distribution of Eu-mtb particles in MMMs. As shown in Supplementary Figure S2A–C, the fluorine element from PVDF and the europium element from Eu-mtb are both distributed uniformly in the Eu-mtb MMM (70 wt%), which confirms the well distribution of Eu-mtb particles in the PVDF matrix. Furthermore, the pure PVDF membrane is white and transparent; with the increase in the loading of white-powder Eu-mtb , the transparency of MMMs decreases gradually, which further demonstrates the successful incorporation and uniform distribution of Eu-mtb particles in the PVDF matrix (Supplementary Figure S3). The photograph of the Eu-mtb MMM (70 wt%) is shown in Supplementary Figure S2D. It can be seen that the membrane is free of macroscopic defects and has the characteristic of flexibility at the same time, making it suitable for practical sensing application.

As a fluorescent sensor for the removal and detection of Cr_2_O_7_^2-^ within industrial waste water, the thermal and acid–base stability are of great importance. The thermal and chemical stability of the Eu-mtb MMM (70 wt%) were investigated by immersing the membrane into aqueous solutions with different temperatures from 40 to 80°C and various pH values from 3.39 to 7.88. As shown in Supplementary Figure S4,5, the PXRD patterns under the aforementioned conditions for 8 h are identical to that of the original Eu-mtb MMM (70 wt%), indicating the excellent chemical and thermal stability of the Eu-mtb MMM (70 wt%), which makes it competent for the removal and detection of Cr_2_O_7_^2−^ under environmentally relevant conditions.

### 3.2 Adsorption Behaviors of Cr_2_O_7_^2−^ by Eu-mtb MMMs

**FIGURE 3:**
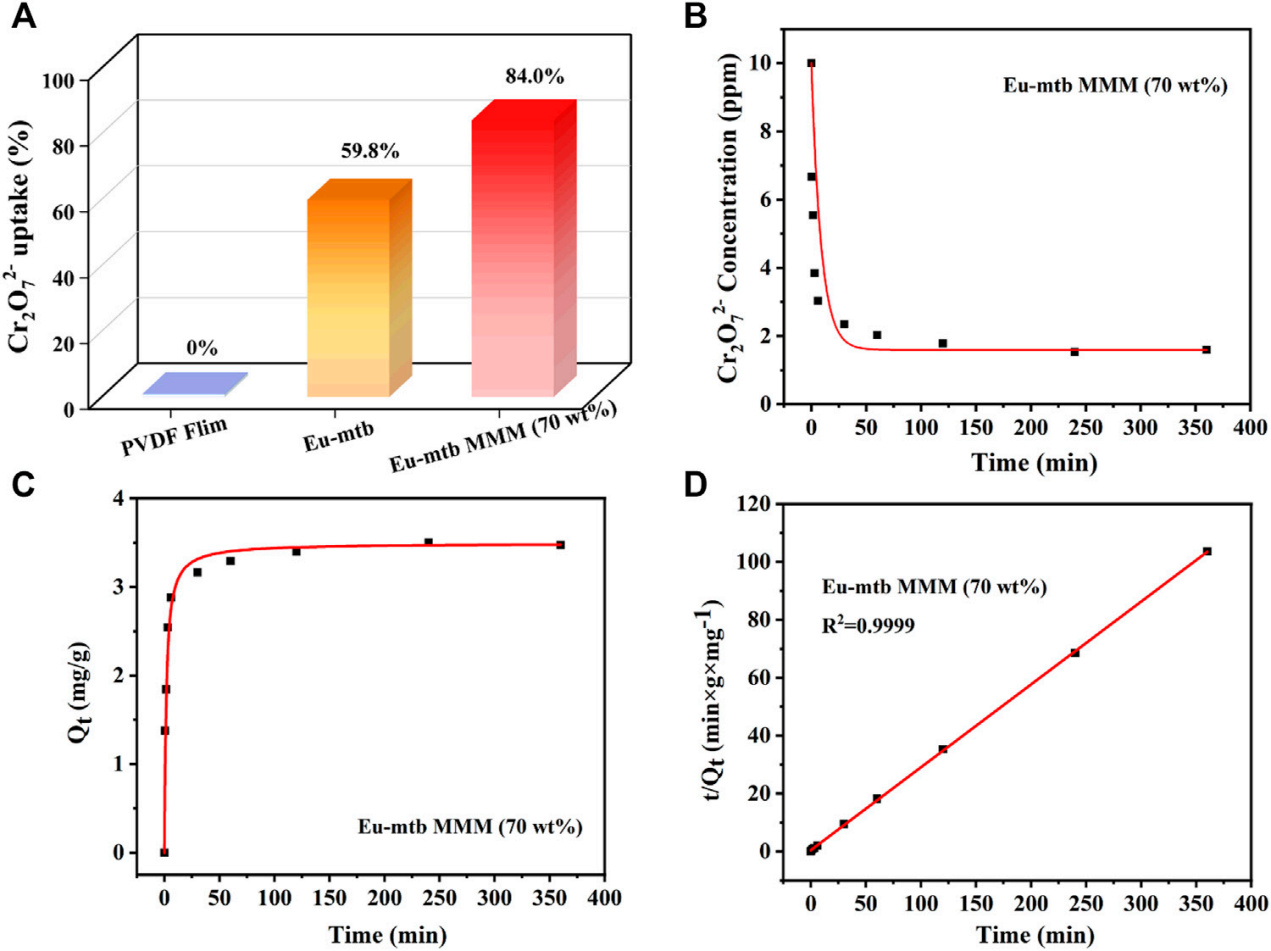
**(A)** Cr_2_O_7_^2−^ removal ability of powder-form Eu-mtb and MMMs with different Eu-mtb weight loadings (0 and 70 wt%). **(B)** Effect of time on the residual concentrations of Cr_2_O_7_^2−^ at different times. **(C)** Effect of time on the amount of Cr_2_O_7_^2−^ adsorbed by the Eu-mtb MMM (70 wt%) at different times. **(D)** Corresponding linear fitting of the adsorption kinetics *via* the pseudo–second-order model.

Adsorption kinetics was investigated first to evaluate the removal efficiency of Cr_2_O_7_^2−^ over Eu-mtb MMMs. The effect of the contact time on the adsorption of Cr_2_O_7_^2-^ from aqueous solution is shown in Figure 3. The pure PVDF membrane displays no uptake of Cr_2_O_7_^2−^ (Eq. 1, Supporting information) after being immersed in the aqueous solution of Cr_2_O_7_^2−^ for 24 h, indicating that the polymer in MMMs has no effect on the removal properties. With the increased loading of Eu-mtb in MMMs, the uptake of Cr_2_O_7_^2−^ increases gradually (Supplementary Figure S6). When the loading of Eu-mtb reaches 70 wt%, the residual concentrations of Cr_2_O_7_^2−^ decreases significantly with immersion time, and around 84.0%, Cr_2_O_7_^2−^ can be removed from the aqueous solution after 24 h, whereas the uptake of Cr_2_O_7_^2−^ by powder-form Eu-mtb is only 59.8% (Figures 3A,B). Therefore, the Eu-mtb MMM (70 wt%) was selected for the following adsorption experiments. In order to explore the adsorption mechanism, the kinetic data of the Eu-mtb MMM (70 wt%) were fitted with the pseudo–second-order kinetic model (Eq. 2, Supporting information). As shown in Figures 3C,D, an extremely high correlation coefficient (*R* ^2^ = 0.9999) was obtained, suggesting that the adsorption kinetics of Cr_2_O_7_^2−^ follow well with the pseudo–second-order model. The calculated half-adsorption time t_1/2_ (Eq. 4, Supporting information) of the Eu-mtb MMM (70 wt%) is 1.62 min, indicating the quick response to Cr_2_O_7_^2−^ in aqueous solutions.

**FIGURE 4:**
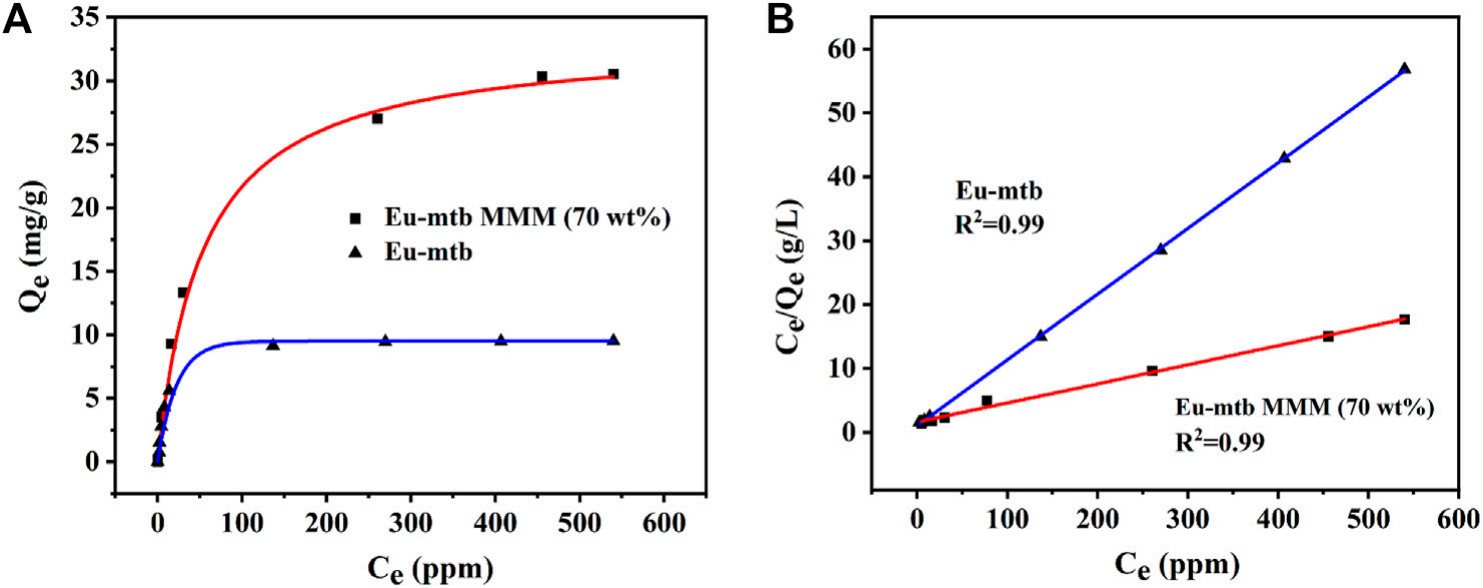
**(A)** Adsorption isotherms for Cr_2_O_7_^2−^ over the Eu-mtb MMM (70 wt%) and powder-form Eu-mtb. **(B)** Corresponding linear fitting curve of the Langmuir isotherm model.

To further confirm the adsorption capacities of the Eu-mtb MMM (70 wt%) toward Cr_2_O_7_^2−^, the adsorption isotherms were carried out. The adsorption capacities of the Eu-mtb MMM (70 wt%) depend on the initial concentrations of Cr_2_O_7_^2−^ water solutions. Therefore, Cr_2_O_7_^2−^ solutions with various concentrations were used to determine the adsorption capacities. As shown in Figure 4, the adsorption isotherms of Cr_2_O_7_^2−^ could be well fitted using the Langmuir model (Eq. 5, Supporting information). The overall adsorption capacity of the Eu-mtb MMM (70 wt%) for Cr_2_O_7_^2−^ reaches up to 33.34 mg/g, which is 3.4 times that of the powder-form Eu-mtb (9.7 mg/g) and higher than some reported MOF-based adsorbents (Supplementary Table S1). It is speculated that the significantly enhanced adsorption rate and capacity of the Eu-mtb MMM (70 wt%) might be attributed to the synergy of free volume at the interface between PVDF and Eu-mtb particles, permanent porosity of Eu-mtb, and the electrostatic interaction between Cr_2_O_7_^2−^ and the cationic framework, facilitating the enrichment of Cr_2_O_7_^2−^ inside MMMs (Supplementary Figure S7).

**FIGURE 5:**
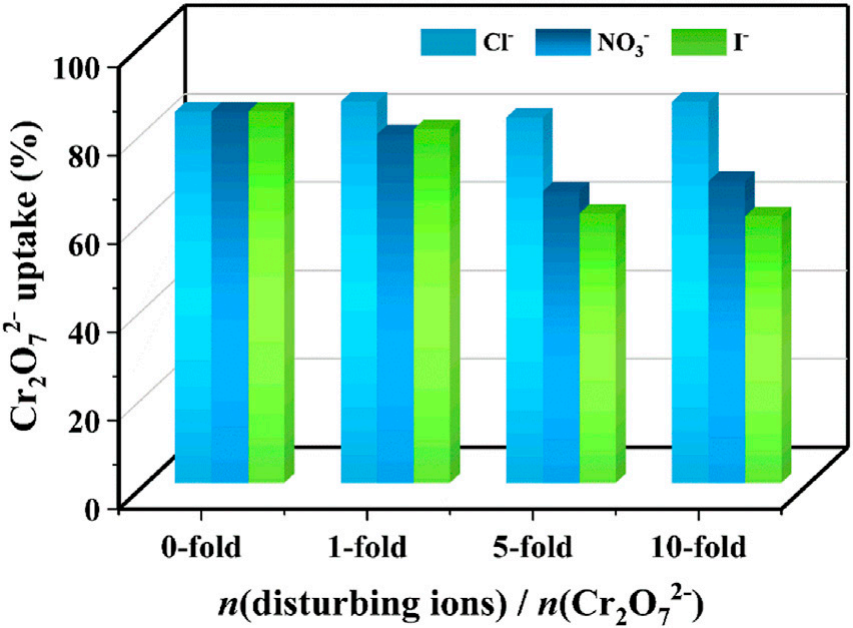
Effect of the disturbing ions (Cl^−^, NO_3_^−^ and I^−^) on the Cr_2_O_7_^2−^ adsorption ability over the Eu-mtb MMM (70 wt%).

Considering that various anions frequently coexist with Cr_2_O_7_^2−^ in environment-related conditions, it is of vital importance to investigate the effect of competing ions on the sorption ability of the Eu-mtb MMM (70 wt%) toward Cr_2_O_7_^2−^. As shown in Figure 5, the change of Cr_2_O_7_^2−^ uptake was negligible even when there was a 10-fold excess mole of Cl^−^. When there were 10-fold excess moles of NO_3_^−^ and I^−^, the uptake of Cr_2_O_7_^2−^ was still at a high efficiency of 60.4%, indicating that the Eu-mtb MMM (70 wt%) exhibits high selectivity to these environmental pollutants.

### 3.3 Fluorescence Sensing of Cr_2_O_7_^2−^ by Eu-mtb MMMs

By virtue of the massive enrichment of Eu-mtb inside MMMs, the sufficient interactions between Cr_2_O_7_^2−^ and the framework can be achieved, which is of great benefit for enhancing the sensing sensitivity towards Cr_2_O_7_^2−^ in aqueous media. Therefore, the fluorescence sensing of Cr_2_O_7_^2−^ by Eu-mtb MMMs is investigated in detail therewith. As shown in Supplementary Figure S8, the solid-state fluorescence measurements showed that H_4_mtb exhibits an intense broad band with a maximum at 416 nm upon excitation at 335 nm, whereas Eu-mtb exhibits characteristic Eu^3+^ emissions with the strongest emission peak at 616 nm from the 5D_0_→^7^F_2_ induced by the electronic dipole transition upon excitation at 280 nm. The absence of the emission peak of the ligand in Eu-mtb indicates that the ligand can sensitize the luminescent Eu^3+^ effectively through the “antenna effect” process.

The emission spectra of Eu-mtb MMMs with different weight loadings were investigated upon 280 nm UV radiation (Supplementary Figure S9). The pure PVDF membrane displays no fluorescence, indicating that the polymer in MMMs has no effect on the sensing test of Cr_2_O_7_^2−^. With the increased loading of Eu-mtb in MMMs, the characteristic emission peak of Eu-mtb appears and increases, reaching maximum when the loading of Eu-mtb is 70 wt%. Therefore, the Eu-mtb MMM (70 wt%) was selected for the following fluorescence sensing of Cr_2_O_7_^2−^. Furthermore, it can be seen that the Eu-mtb MMM (70 wt%) exhibits fluorescence intensity similar to powder-form Eu-mtb under the same experiment conditions, indicating that Eu-mtb doping with membranes almost has no effect on the fluorescence intensity of Eu-mtb particles (Supplementary Figure S10). As a fluorescent sensor for the detection of Cr_2_O_7_^2−^ within water, the fluorescence stability is a factor that should be concerned. As shown in Supplementary Figures S11,12, the emission intensity of Eu-mtb MMMs (70 wt%) at 616 nm under excitation at 280 nm shows negligible changes with the varied temperatures and pH values, which provides a prerequisite for the application of the Eu-mtb MMM (70 wt%) as a fluorescent sensor in the aqueous environment. In order to demonstrate the feasibility of as-prepared MMMs as a platform for sensitive fluorescence sensing, the Eu-mtb MMM (70 wt%) was employed to detect Cr_2_O_7_^2-^ in aqueous solutions by the flow-through and soaking method (soaking for 8 h). As shown in Supplementary Figure S13, the fluorescence quenching of Eu-mtb MMMs (70 wt%) is more significant upon treatment with Cr_2_O_7_^2−^ aqueous solution (10^–3^ M, 100 ml) by the flow-through method, demonstrating that the flow-through method is preferable to conduct the following Cr_2_O_7_^2−^ sensing experiments. The time that has been consumed by the flow-through method is nearly 30 min, which is significantly shorter than the soaking method. Furthermore, as shown in Supplementary Figure S14, the repeated experiments of the Eu-mtb MMM (70 wt%) for Cr_2_O_7_^2−^ by the flow-through method indicate that this method is reliable and resultful.

**FIGURE 6:**
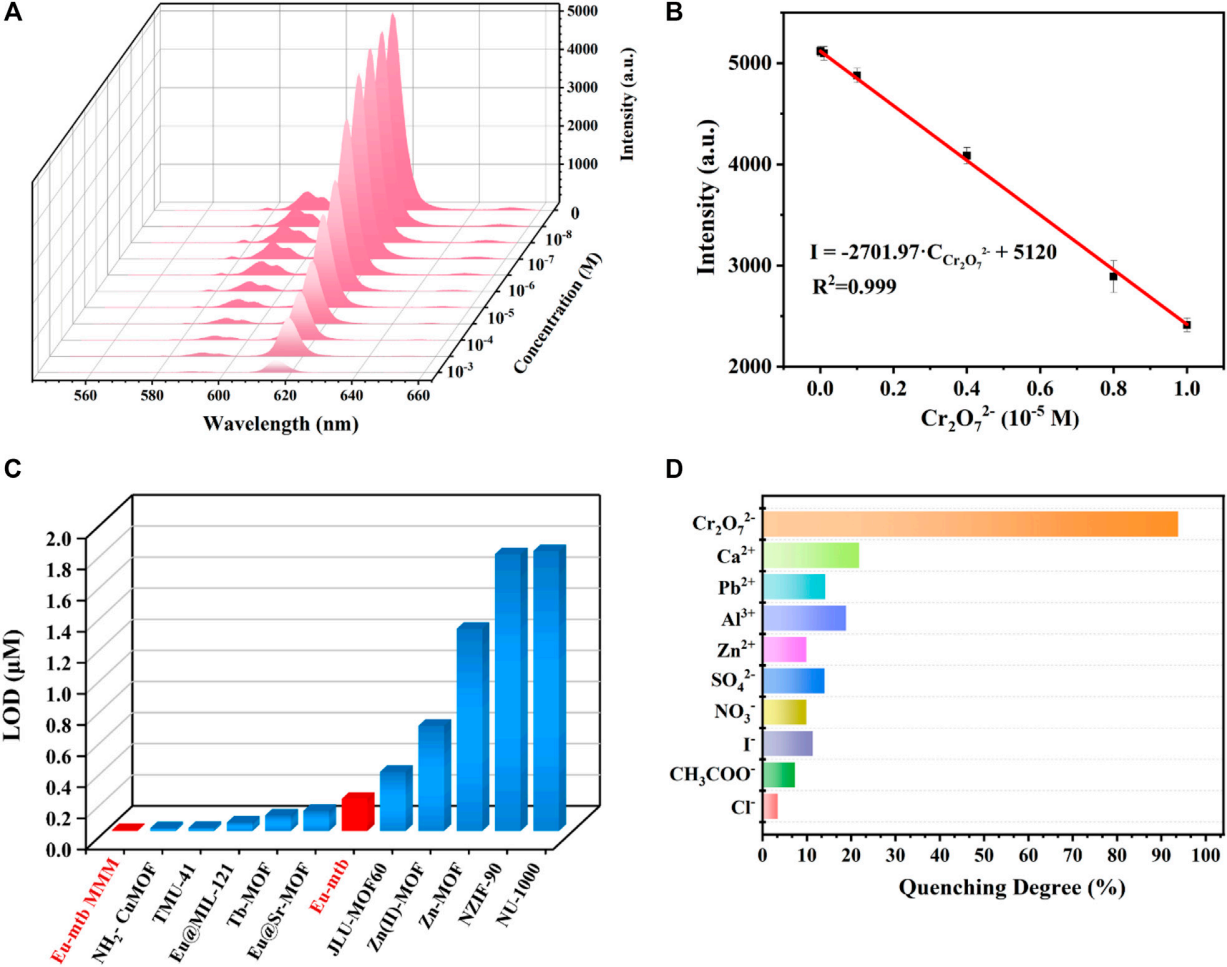
**(A)** Emission spectra of the Eu-mtb MMM (70 wt%) with increasing concentrations (0–10^–3^ M) of Cr_2_O_7_^2-^. **(B)** Linear relationship (0–10^–5^ M) of the emission intensity of the Eu-mtb MMM (70 wt%) quenched by Cr_2_O_7_^2−^. **(C)** The limit of detection of reported MOFs for the fluorescence sensing of Cr_2_O_7_^2−^. **(D)** The fluorescence quenching degree of the Eu-mtb MMM (70 wt%) at 616 nm toward different aqueous solutions of various anions and cations (100 ml 10^–3^ M).

To evaluate the sensing property of the Eu-mtb MMM (70 wt%), the fluorescence of the Eu-mtb MMM (70 wt%) was monitored upon treatment with different concentrations of Cr_2_O_7_^2−^ aqueous solutions (100 ml) by the flow-through method. As shown in Figure 6A and Supplementary Figure S15, the emission intensity of the Eu-mtb MMM (70 wt%) gradually decreased with the increasing concentration of Cr_2_O_7_^2–^ from 0 to 10^–3^ M. When the concentration of Cr_2_O_7_^2–^ approached 10^–3^ M, the fluorescence signal of the Eu-mtb MMM (70 wt%) was almost completely quenched. In the range of 1–10 mM (Figure 6B), the luminescence intensity and concentration of Cr_2_O_7_^2–^ exhibited a good linear relationship with a correlation shown by the equation $I_{616}\;=\;-2701.97\cdot C\;\left({Cr}_{2}O_{7}{\;}^{2-}\right)\;+\;5120$ (*R* ^2^ = 0.999), in which *I*_616_ represents the luminescence intensity of the Eu-mtb MMM (70 wt%) at *λ* = 616 nm. The limit of detection (LOD) was calculated according to the following equations (Zhang et al., 2018):

$$\delta =\sqrt{\frac{\sum {\left(F_{0}-F_{1}\right)}^{\;2}}{N-1}},$$

$$LOD=\frac{3\delta }{S}.$$

where *δ* is the standard deviation calculated from blank measurements of the probe (Eu-mtb MMM (70 wt%)), and *S* is the slope value obtained from the linear fit in the low-concentration region. The LOD value of the Eu-mtb MMM (70 wt%) toward Cr_2_O_7_^2–^ was calculated to be 5.73 nM. In order to verify MMMs have superior sensing properties than powder-form Eu-mtb, the sensing experiments of powder-form Eu-mtb were conducted according to published literatures. In brief, 1 mg finely ground Eu-mtb powders were dispersed into aqueous solutions of Cr_2_O_7_^2–^ (2 ml) with various concentrations, which were then treated by ultrasonication to form a homogenous suspension before fluorescence measurement. As shown in Supplementary Figure S16,17, in the range of 1–10 mM, the luminescence intensity and concentration of Cr_2_O_7_^2–^ exhibited a good linear relationship with a correlation shown by the equation $I_{616}\;=\;-2045.96\cdot C\;\left({Cr}_{2}O_{7}{\;}^{2-}\right)\;+\;8001$ (*R* ^2^ = 0.97). The LOD value of the Eu-mtb powder toward Cr_2_O_7_^2–^ was calculated to be 0.207 mM, inconsistent with what has been reported in the literature (Liu et al., 2017), which is probably due to the different evaluation formulae of the LOD. It is noteworthy that the LOD value of the Eu-mtb MMM (70 wt%) is significantly lower than that of Eu-mtb and other reported fluorescence powder-form MOFs for Cr_2_O_7_^2–^ (Figure 6C) (Hao and Yan, 2016; Lin et al., 2017; Jin et al., 2018; Xiao et al., 2018; Abdollahi and Morsali, 2019; Liu et al., 2019; Mi et al., 2019; Wiwasuku et al., 2019; Guo et al., 2020; Qiu et al., 2021), indicating that the Eu-mtb MMM (70 wt%) is a promising luminescent platform for sensing Cr_2_O_7_^2–^ in aqueous solution with excellent sensitivity. It is speculated that the dramatically enhanced sensitivity of the Eu-mtb MMM (70 wt%) might be attributed to the enrichment of Cr_2_O_7_^2–^ from the aqueous solutions inside MMMs and the uniform distribution of Eu-mtb particles without aggregation in MMMs, making it possible for the sufficient interactions between Cr_2_O_7_^2–^ and the Eu-mtb framework. Given the fact that there are a variety of interfering ions within industrial waste water, the selectivity of the Eu-mtb MMM (70 wt%) toward Cr_2_O_7_^2–^ was investigated. As shown in Figure 6D, only Cr_2_O_7_^2–^ significantly quenched the fluorescence intensity of Eu-mtb MMMs (70 wt%), while other cations (Ca^2+^, Pb^2+^, Al^3+^, and Zn^2+^) and anions (SO_4_^2-^, NO_3_^−^, I^−^、CH_3_COO^−^ and Cl^−^) exhibit negligible effects on the fluorescence intensities of the Eu-mtb MMM (70 wt%) sample. The different degrees of quenching effects on the fluorescence intensities imply that the Eu-mtb MMM (70 wt%) could be considered as a potential candidate for the selective probing of Cr_2_O_7_^2−^. In addition, as shown in Supplementary Figure S18, the as-prepared MMM is insoluble and stable in various chemical aqueous solutions, which further indicates the possibility of the Eu-mtb MMM (70 wt%) to detect Cr_2_O_7_^2−^in a chemical environment.

### 3.4 Mechanism for Sensing Cr_2_O_7_^2−^

**FIGURE 7:**
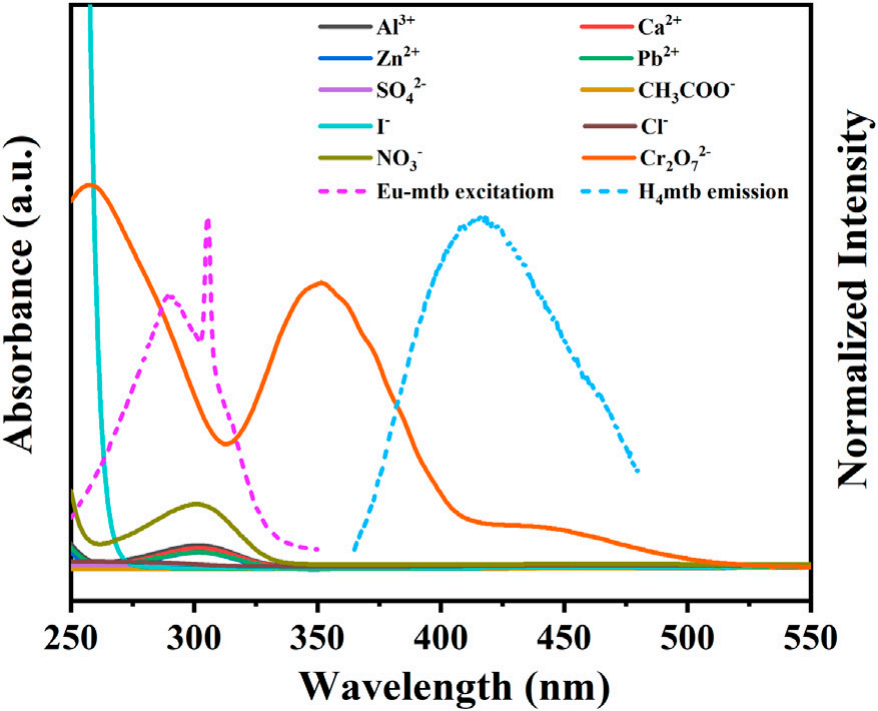
UV-vis spectra of different aqueous solutions of various ions and fluorescent spectra of Eu-mtb and H_4_mtb.

In order to explore the possible mechanism of the fluorescence quenching from the Eu-mtb MMM (70 wt%) induced by Cr_2_O_7_^2−^, UV-vis spectra of aqueous solutions of various anions and cations were investigated. As shown in Figure 7, it is notable that the excitation peak of Eu-mtb is partially overlapped by the strong absorption of Cr_2_O_7_^2−^, resulting in the inner filter effect (IFE) (Mi et al., 2019; Guo et al., 2020). The competition of excitation light between the host material and Cr_2_O_7_^2−^ led to the selective fluorescence quenching response of the Eu-mtb MMM (70 wt%). Apart from this, it is evident that the emission peak of H_4_mtb is also partially overlapped by the absorption of Cr_2_O_7_^2−^, while no overlap between the emission band of H_4_mtb and the absorption bands of other ions is observed. Therefore, the resonance energy transfer from H_4_mtb to Cr_2_O_7_^2−^ might take place, and the “antenna effect” process from H_4_mtb to Eu^3+^ is inhibited to some extent (Dong Z.-P. et al., 2020). Based on these pieces of evidence, both competitive absorption and resonance energy transfer are responsible for the fluorescence quenching of the Eu-mtb MMM (70 wt%) toward Cr_2_O_7_^2−^.

## 4 Conclusion

In summary, MOF-based MMMs have been evaluated as a candidate for the removal and detection of Cr_2_O_7_^2-^ in aqueous media simultaneously. The higher adsorption capacity (33.34 mg/g) and lower LOD value (5.73 nM) of the as-prepared Eu-mtb MMM (70 wt%) than powder-form Eu-mtb for Cr_2_O_7_^2−^ are attributed to the enrichment of Cr_2_O_7_^2−^ inside MMMs and the uniform distribution of cationic Eu-mtb particles within MMMs, which facilitate the interactions between Cr_2_O_7_^2−^ and the Eu-mtb framework sufficiently. Furthermore, the combination of the flexibility of the polymer and the excellent properties of MOFs provides the possibility to overcome the disadvantages of powder-form MOFs and pure MOF membranes. Therefore, MOF-based MMMs is a functional and promising platform for a wide range of practical applications.

## Data Availability

The original contributions presented in the study are included in the article/Supplementary Material, further inquiries can be directed to the corresponding authors.
