# Supplementary material for: Cationic Metal–Organic Framework-Based Mixed-Matrix Membranes for Fast Sensing and Removal of Cr2O7 2− Within Water
Source: Front Chem. 2022 Feb 28;10:852402. doi: 10.3389/fchem.2022.852402 (PMC8918786; doi:10.3389/fchem.2022.852402)
Supplement: Supplementary file 1 [file DataSheet1.DOCX]

Supplementary Material

**Metal-Organic Framework Based Mixed-Matrix Membranes for Fast Sensing and Removal of Cr_2_O_7_^2-^ within water**

# Adsorption Experiments

**Adsorption Kinetics:** 2.1 mL of mixed solutions were cast onto a glass plate to fabricate membranes. During the adsorption process, the as-prepared MMMs were used for the removal of Cr_2_O_7_^2-^ with the concentration of 10 ppm. The Cr_2_O_7_^2-^ solutions (48 mL) containing the MMMs were mixed well with magnetic stirring for 6 h at 25 ℃. During the stirring period, 2 mL of the mixture was taken out and filtered by syringe filters (PTFE, 0.25 μm), and the residual concentration of Cr_2_O_7_^2-^ in the supernatant liquid was evaluated by UV-vis absorbance (at λ = 257 nm). Corresponding parameters were calculated according to the following formula ([Zhu et al., 2020](#_ENREF_1)).

The Cr_2_O_7_^2-^ uptake (%) was calculated according to the equation (1):

$$\text{ }\text{ Cr}\text{2}\text{O}\text{7}\text{2- }\text{uptake (\%) }\text{=}\text{ }\frac{\text{C}_{\text{0}}\text{-}\text{C}_{\text{e}}}{\text{C}_{\text{0}}}\text{ × 100\% }\text{ }\text{(1)}$$

Where *C_0_* and *C_e_* are the initial and equilibrium concentrations of Cr_2_O_7_^2-^ (mg∙L^-1^) in the solution, respectively.

The pseudo second-order equation can be expressed by equation (2):

$$\frac{\text{t}}{\text{Q}_{\text{t}}}\text{ }\text{=}\text{ }\frac{\text{1}}{\text{k}_{\text{2}}\text{Q}_{\text{e}}^{\text{2}}}\text{ + }\frac{\text{t}}{\text{Q}_{\text{e}}}\text{ (2)}$$

Where *Q_t_* and *Q_e_* are the amount of Cr_2_O_7_^2-^ (mg∙g^-1^) onto MMMs at time *t* and the equilibrium (min), respectively. *k_2_* represents the equilibrium rate constant of pseudo second-order equation (g∙mg^−1^∙min^−1^).

The adsorption capacity *Q_t_* was calculated by equation (3):

$$\text{ }\text{Q}_{\text{t}}\text{ }\text{=}\text{ }\frac{{\text{(}\text{C}}_{\text{0}}\text{-}\text{C}_{\text{t}}\text{)}\text{V}}{\text{m}}\text{ (3)}$$

Where *C_t_* is the concentration of Cr_2_O_7_^2-^ (mg∙L^-1^) at time *t*. *V* is the total volume of Cr_2_O_7_^2-^ solution (L), and *m* is the mass of Eu-mtb in MMMs (g).

Based on the pseudo second-order kinetic model, the half-adsorption time *t_1/2_* (min) were calculated according to equation (4):

$$\text{ }\text{t}_{\text{1/2}\text{ }}\text{ =}\text{ }\frac{\text{1}}{\text{k}_{\text{2}}\text{Q}_{\text{e}}}\text{ (4)}$$

**Adsorption Isotherm:** 265 μL of dope solutions were cast onto a glass plate to fabricate membranes. To obtain the adsorption capacity, MMMs were dispersed in 10 mL of Cr_2_O_7_^2-^ solutions with a known concentration between 10 and 600 ppm, respectively. The mixtures were stirred at 25 °C for 24 hours and then filtered by syringe filters (PTFE, 0.25 μm), and the residual concentrations of Cr_2_O_7_^2-^ were evaluated by UV-vis spectroscopy. The theoretical maximum adsorption capacity *Q_m_* (mg∙g^-1^) was calculated according to equation (5):

$$\frac{\text{C}_{\text{e}}}{\text{Q}_{\text{e}}}\text{ =}\text{ }\frac{\text{C}_{\text{e}}}{\text{Q}_{\text{m}}}\text{ + }\frac{\text{1}}{\text{Q}_{\text{m}}\text{k}_{\text{L}}}\text{ }\text{ }\text{ (5)}$$

Where *k_L_* represents the constant of Langmuir model (L∙mg^-1^).

# Supplementary Figures


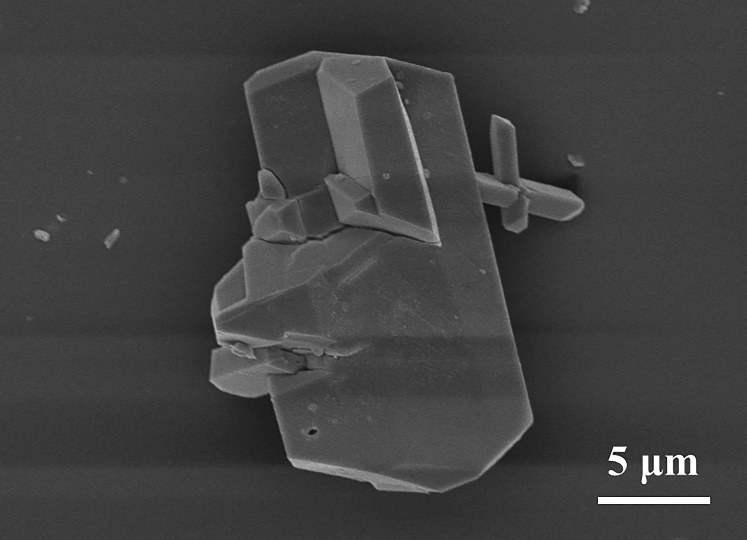


**Supplementary Figure 1**. SEM image of Eu-mtb.


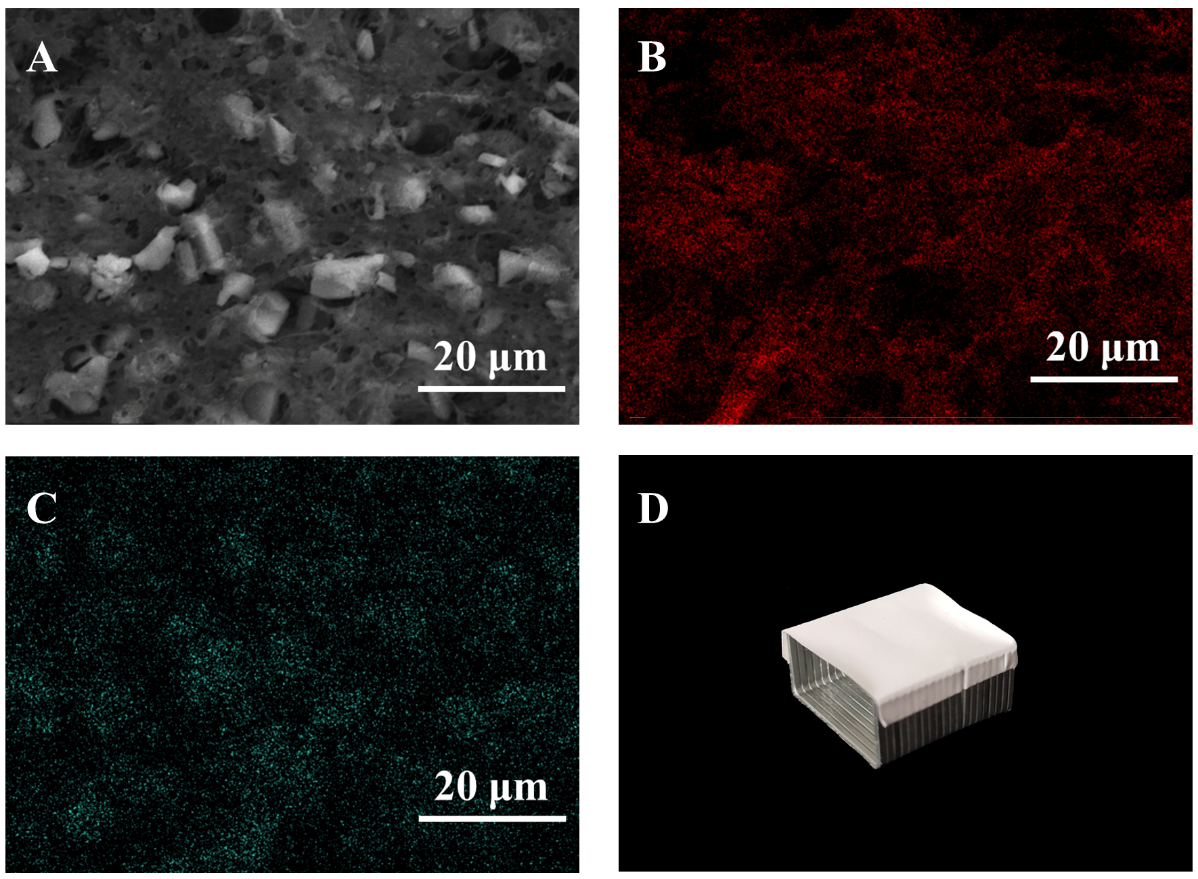


**Supplementary Figure 2.** SEM image of Eu-mtb. SEM image of **(A)** Eu-mtb MMM (70 wt%) and the elemental mapping images of **(B)** fluorine and **(C)** europium. **(D)** Photograph of Eu-mtb MMM (70 wt%).


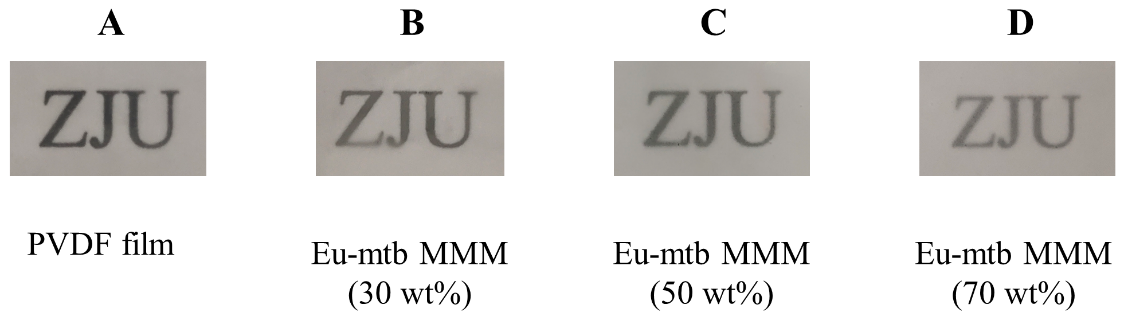


**Supplementary Figure 3.** The transparency of (A) pure PVDF film, (B) Eu-mtb MMM (30 wt%), (C) Eu-mtb MMM (50 wt%) and (D) Eu-mtb MMM (70 wt%).





**Supplementary Figure 4.** PXRD patterns of Eu-mtb MMM (70 wt%) samples soaked in aqueous solution with different temperatures for 8 h.





**Supplementary Figure 5.** PXRD patterns of Eu-mtb MMM (70 wt%) samples soaked in aqueous solution with different temperatures for 8 h.





**Supplementary Figure 6.** The Cr_2_O_7_^2-^ adsorption ability of Eu-mtb MMMs with different Eu-mtb weight loadings (0, 30, 50 and 70 wt%).


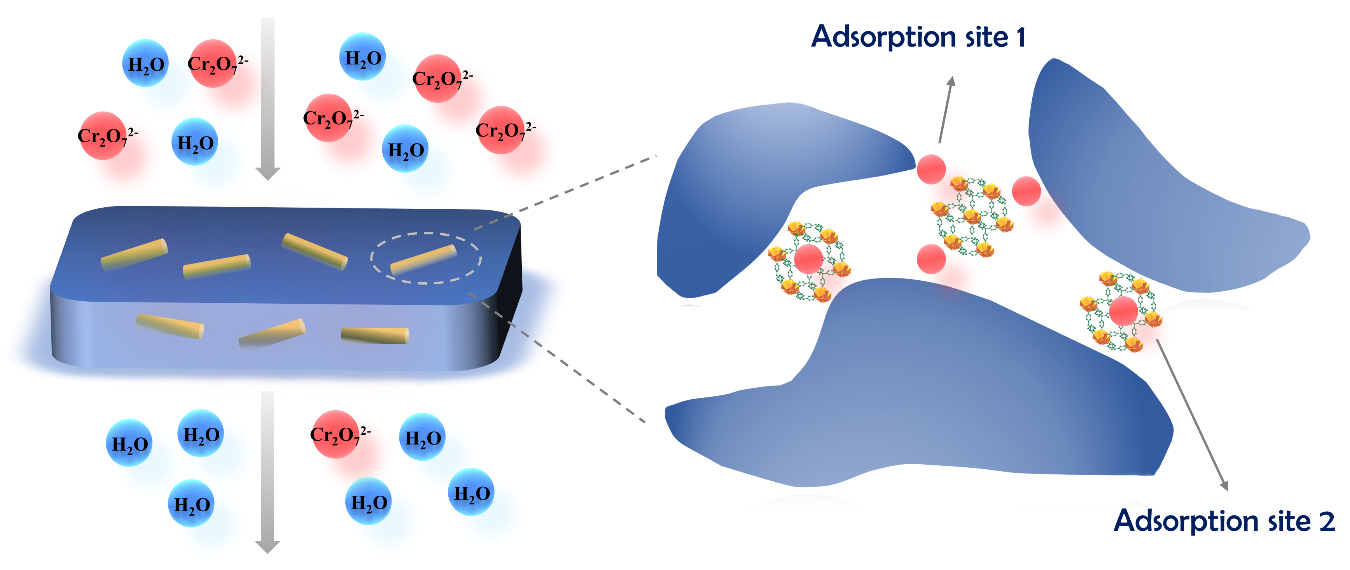


**Supplementary Figure 7.** The scheme of the removal process of Cr_2_O_7_^2-^ by Eu-mtb MMMs.





**Supplementary Figure 8.** Excitation (purple line), emission (blue line) spectra of H_4_mtb and emission (red line) spectra of Eu-mtb (λ_ex_=280 nm) in solid state.





**Supplementary Figure 9.** The emission spectra of PVDF membrane, Eu-mtb MMM (30 wt%), Eu-mtb MMM (50 wt%) and Eu-mtb MMM (70 wt%) (λ_ex_=280 nm).





**Supplementary Figure 10.** The fluorescence intensity of powder-form Eu-mtb and Eu-mtb MMM (70 wt%).





**Supplementary Figure 11.** The spectral stability of Eu-mtb MMM (70 wt%) soaked in aqueous solution with different temperatures.





**Supplementary Figure 12.** The spectral stability of Eu-mtb MMM (70 wt%) soaked in aqueous solution with different pH values.





**Supplementary Figure 13.** The emission spectra of Eu-mtb MMM (70 wt%) upon Cr_2_O_7_^2-^ (100 mL10^-3^M) treatment with the flow-through method and soaking method, compared with Eu-mtb MMM (70 wt%).





**Supplementary Figure 14.** The Repeated experiments of the fluorescent quenching degree of Eu-mtb MMM (70 wt%) upon treatment with Cr_2_O_7_^2-^ aqueous solution (10^-3^ M, 100 mL) by the flow-through method.





**Supplementary Figure 15.** Concentration dependence (0~10^-4^ M) of the emission intensity of Eu-mtb MMM (70 wt%) quenched by Cr_2_O_7_^2-^ at 616 nm.





**Supplementary Figure 16.** The emission spectra of powder-form Eu-mtb with increasing concentrations (0~10^-4^ M) of Cr_2_O_7_^2-^.





**Supplementary Figure 17.** Linear relationship (0~10^-5^ M) of the emission intensity of Eu-mtb quenched by Cr_2_O_7_^2-^.





**Supplementary Figure 18.** PXRD patterns of Eu-mtb MMM (70 wt%) toward different aqueous solutions of various anions and cations (100mL 10^-3^ M).

**Table S1** Comparison of Cr_2_O_7_^2-^ adsorption ability of Eu-mtb MMM (70 wt%) with other MOFs.

| **MOF based**  **Adsorbents** | **Maximum Capacity**  **(mg/g)** | **Ref.** |
| --- | --- | --- |
| Eu-mtb | 9.7 | *ACS Appl. Mater. Interfaces.* 2017, 9, 16448-16457 |
| **Eu-mtb MMM (70 wt%)** | **33.34** | **This work** |
| Zr-MOF-3 | 30 | *J. Mater. Chem. C.* 2020, 8, 16974-16983 |
| NU-1000 | 76.8 | *Inorg. Chem.* 2017, 56**,** 14178-14188 |
| MIL-100(Fe) | 30.45 | *J. Solid State Chem.* 2020, 291, 121636 |
| UPC-50 | 56.8 | *Mater. Chem. Front.* 2020, 4, 1150-1157 |
| FIR-53 | 74.2 | *Chem. Mater.* 2015, 27, 205-210 |
| MOF-867 | 53.4 | *Chem. Commun.* 2015, 51**,** 14732-14734 |
| PCN-134 | 57 | *J. Am. Chem. Soc.* 2016, 138, 6636-6642 |

# Reference

Zhu, K., Fan, R., Wu, J., Wang, B., Lu, H., Zheng, X., et al. (2020). MOF-on-MOF Membrane with Cascading Functionality for Capturing Dichromate Ions and p-Arsanilic Acid Turn-On Sensing. *ACS Appl. Mater. Interfaces* 12(52)**,** 58239-58251. doi: 10.1021/acsami.0c17875.
